# Saliva as Biomarker for Oral and Chronic Degenerative Non-Communicable Diseases

**DOI:** 10.3390/metabo13080889

**Published:** 2023-07-27

**Authors:** Michele Basilicata, Massimo Pieri, Giulia Marrone, Eleonora Nicolai, Manuela Di Lauro, Vincenza Paolino, Flaminia Tomassetti, Ilaria Vivarini, Patrizio Bollero, Sergio Bernardini, Annalisa Noce

**Affiliations:** 1 UOSD Special Care Dentistry, Policlinico Tor Vergata, 00133 Rome, Italy; 2 Department of Experimental Medicine and Surgery, University of Rome Tor Vergata, 00133 Rome, Italy; 3 Department of Laboratory Medicine, “Tor Vergata” University Hospital, Viale Oxford 81, 00133 Rome, Italy; 4 Department of Systems Medicine, University of Rome Tor Vergata, 00133 Rome, Italy; 5 UOSD Nephrology and Dialysis, Policlinico Tor Vergata, 00133 Rome, Italy

**Keywords:** saliva, chronic degenerative non-communicable diseases, chronic kidney disease, oral diseases, uremic toxins, gut microbiota, oral microbiota, salivary biomarkers

## Abstract

Saliva is a very complex fluid and it is essential to maintain several physiological processes and functions, including oral health, taste, digestion and immunological defenses. Saliva composition and the oral microbiome can be influenced by several factors, like diet and smoking habits, and their alteration can represent an important access point for pathogens and, thus, for systemic illness onset. In this review, we explore the potentiality of saliva as a new tool for the early detection of some pathological conditions, such as oral diseases, chronic degenerative non-communicable diseases, among these chronic kidney disease (CKD). We also examined the possible correlation between oral and systemic diseases and oral and gut microbiota dysbiosis. In particular, we deeply analyzed the relationship between oral diseases and CKD. In this context, some salivary parameters can represent a new device to detect either oral or systemic pathologies. Moreover, the positive modulation of oral and gut microbiota induced by prebiotics, postbiotics, or symbiotics could represent a new possible adjuvant therapy in the clinical management of oral diseases and CKD.

## 1. Introduction

The importance of saliva as a biological fluid is often neglected, but saliva is involved in several physiological processes, including the perception of oral taste, digestion and immunological defenses [1]. In fact, saliva is a very complex fluid and its composition is influenced by many factors related to the physiological status of the body. Thus, it represents a new challenge from a clinical point of view.

Saliva is the product of salivary gland secretion, which is regulated by both the sympathetic and parasympathetic systems and once poured into the oral cavity, saliva is mixed with liquids secreted by the buccal epithelial cells, cellular infiltrate and microorganisms [2]. It is rich in minerals, electrolytes (calcium, zinc and magnesium), hormones (adrenomedullin), enzymes (α-amylase), immunoglobulins, cytokines, antimicrobial peptides (AMP), glycoprotein (lactoferrin), mucins and oral tissue repairs (epidermal growth factor and histatins) [3]. Hence, the analysis of saliva and the study of the salivary metabolic profile can represent a new useful tool in the diagnosis and prognosis of chronic degenerative non-communicable diseases (NCDs), including those not related to oral health. 

Several factors influence and induce modifications in saliva composition and the oral microbiome, which is one of the most balanced ecosystems after the gut microbiome. The oral cavity in a healthy individual can contain more than 300 species of microorganisms [4], including bacteria, fungi, viruses, archaea and protozoa, even if aerobes are mainly present [5].

Recent studies have shown that the oral microbiota can be influenced by various factors such as diet [6,7] and smoking, which can lead to the growth of one type of bacterial species rather than others. An imbalance in the microbial ecosystem is called dysbiosis. Dysbiosis can lead to the numerical expansion of some potentially pathogenic commensal bacterial species. Their consequent dominance in the niche causes an unhealthy status [8]. Dysbiosis of oral microbes is an impairment that can be observed not only in oral diseases, such as periodontitis, but also in systemic diseases, such as cardiovascular and renal diseases [8].

Furthermore, the oral cavity is an important access point for pathogens. Since the oral microbiome has direct access to respiratory and gastrointestinal systems, oral dysbiosis can be responsible for various pulmonary diseases including pneumonia, lung cancer, etc. [9], as well as inflammatory bowel diseases [10].

In general, saliva composition should be linked to several pathological conditions such as chronic degenerative NCDs [11]. The literature widely demonstrates a correlation between salivary proteomics and oral and systemic diseases. In particular, dysbiosis of the oral microbiome was found to be strictly related to chronic kidney disease (CKD) [12,13], pulmonary diseases, cardiovascular diseases, diabetes mellitus, Alzheimer’s disease, cancer and other metabolic diseases [1,8,14,15].

The aim of this review is to highlight how saliva biomarkers can be exploited as a new potential tool for the early detection of some pathological conditions, such as oral diseases and CKD. Moreover, we point out the close relationship between oral dysbiosis and CKD and how CKD itself can induce or worsen oral diseases, and vice versa.

## 2. Search Methods

**Figure 1:**
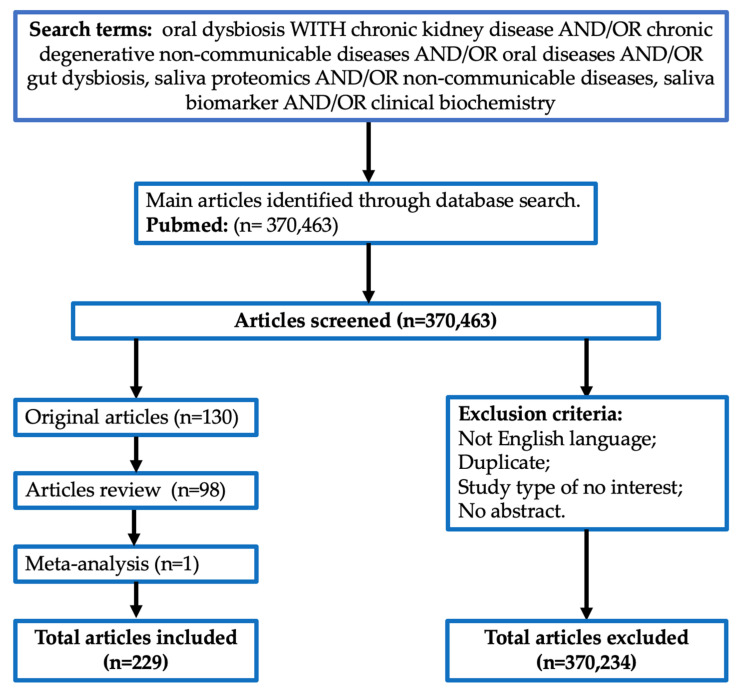
Search methods.

An extensive literature search was performed for articles published up to 30 April 2023 using the Scopus, Web of Science and PubMed online databases. To screen for suitable articles, we included the following keywords, alone or in combination: “oral dysbiosis” with “chronic kidney disease” and/or “chronic degenerative non-communicable diseases” and/or “oral diseases” and/or “gut dysbiosis”, “saliva proteomics” and/or “non-communicable diseases”, and “saliva biomarker” and/or “clinical biochemistry” (Figure 1). All articles included in this review were in the English language and were manually selected by the authors.

## 3. Potential Role of Salivary Biomarkers in the Diagnosis of Chronic Degenerative NCDs

Thanks to noninvasive and relatively easy sampling, saliva has become an increasingly important tool for researchers and clinicians. In fact, up to now, it has been used to detect some pathological conditions, such as infectious diseases, genetic disorders and oral cavity cancer. Moreover, in saliva, biomarkers, represented by different molecule classes including proteome, transcriptome, micro-RNA, metabolome, and microbiome [1], are used for screening purposes in epidemiological studies. The enzyme-linked immunosorbent assay (ELISA), immunofluorometric assay (IFMA), or Luminex assay can be applied to determine salivary concentrations of interleukin (IL)-6, IL-8, tumor necrosis factor (TNF)-α, lysozyme, matrix metalloproteinases (MMP)-8, and tissue inhibitors of metalloproteinases (TIMP)-1. These unspecific inflammatory biomarkers can be used to assess systemic inflammation [16] and the obtained results from an analysis of salivary creatinine and urea can be used as additional CKD diagnostic parameters. To date, recent studies demonstrated a significant correlation between salivary and serum creatinine [17,18,19]. These data helped to establish that saliva is a promising alternative matrix for CKD clinical evaluation. In fact, emerging studies support the diagnostic potential of salivary biomarkers, like urea and creatinine, as a non-invasive tool to estimate renal function.

Several studies in the literature evaluated the performance and transversal utility of salivary biomarkers, not only for CKD but also for a broad spectrum of other pathologies. Moreover, recent studies demonstrated that transcriptomic biomarkers play a role in the noninvasive detection of some types of tumors, as well as resectable pancreatic cancer [20] and breast cancer [21].

Furthermore, a saliva proteomic analysis, including oral squamous cell carcinoma (OSCG) patients, identified transferrin as a potential salivary biomarker for the diagnosis of early-stage OSCG. In particular, the authors noticed that salivary transferrin levels were strongly correlated with tumor size, demonstrating its predictive power [22].

In addition, quantitative assessment of salivary biomarkers can play a key role in the early diagnosis of other chronic degenerative NCDs. A further advantage of saliva analysis is that saliva can be easily and non-invasively collected, and for this reason, saliva could be an ideal biomarker to monitor health status and screen the risk of chronic degenerative NCDs onset. An example of this perspective is a study including Alzheimer’s disease (AD) patients, in which it was determined that the phosphorylated tau/tau ratio in saliva significantly increased compared to healthy controls [23]. The importance of this result is highlighted by noninvasive saliva collection compared to cerebrospinal fluid (CSF) sampling.

Finally, saliva testing can be used to evaluate the health of the oral cavity, including the presence of bacteria and other microorganisms, which can affect overall health or can identify oral diseases such as periodontitis and dental caries [24].

## 4. The Role of Current Salivary Biomarkers in Use and of Oral Microbiota

Nowadays, despite the great number of studies on saliva and the fact that saliva is easy to be collected, its analysis has been used in a limited number of diagnostic applications. Few methods are currently validated for saliva analysis. In fact, many of them are intended for research purposes, instead of diagnostic purposes. Moreover, the available data were collected in studies conducted on a small sample of patients [25,26].

The main applications of salivary biomarkers and the correlations with all their applications are summarized in Table 1.

Furthermore, oral microbiota analysis, with over 700 species of bacteria, plays a fundamental role in the study and in the determination of oral and systemic diseases. For the mouth, the most identifiable bacterial species is *Streptococcus salivarius*, but it also contains bacteria, fungi, viruses and protozoa. 

Nowadays, the methods available to study the microbiome are various and numerous. In short, they include:

- Next generation sequencing (NGS): gene marker analysis (the most used are 16S ribosomal RNA gene sequencing and internal transcribed spacer (ITS)), shogun metagenomics (using untargeted sequencing method), and metatrascriptomics;
- Liquid chromatography–mass spectrometry (LC/CG-MS): metabolomics and metaproteomics.

## 5. Sample Collection for Saliva Proteomics

Saliva turns out to be an easily available matrix but, simultaneously, its collection requires specific considerations and analytical pre-treatment to obtain a suitable sample.

Saliva is a hypotonic fluid secreted by the salivary glands located in the oral cavity. Like all secretions, saliva is primarily composed of water (99%), while only 1% consists of inorganic and organic substances. Salivary secretion is performed by various glands: submandibular, parotid, sublingual and minor salivary glands. 

The fluid secreted by the salivary glands does not always exhibit the same biochemical characteristics. For instance, the quantity of saliva produced by the parotid glands increases significantly after stimuli. Thus, several biochemical differences can be observed between stimulated and unstimulated saliva [70,71,72,73].

Some general differences include:

1. Flow rate: stimulation, such as chewing, talking, or smelling food leads to an increased flow of saliva compared to the resting or unstimulated state. Flow rate affects the concentrations of various salivary components.
2. Water content: unstimulated saliva has a lower water content compared to stimulated saliva, and stimulation triggers the release of larger volumes of watery saliva.
3. Protein composition: the protein composition of saliva can differ between stimulated and unstimulated states. Stimulation typically leads to increased secretion of proteins, such as amylase, mucins and immunoglobulins. These proteins play roles in enzymatic digestion, lubrication and immune defense.
4. Electrolytes: stimulation can affect the concentration of electrolytes in saliva. Stimulated saliva often contains higher levels of electrolytes like sodium, potassium, calcium and bicarbonate compared to unstimulated saliva. These electrolytes are important for maintaining oral pH balance and overall oral health.
5. pH level: stimulation can result in changes in salivary pH. Unstimulated saliva generally has a slightly acidic pH, while stimulated saliva tends to be more neutral or slightly alkaline. The buffering capacity of stimulated saliva helps in neutralizing acids and maintaining a healthier oral environment.
6. Enzymes: stimulation triggers the release of various enzymes in saliva. For example, stimulated saliva contains higher levels of alpha-amylase, which initiates the digestion of carbohydrates. Other enzymes, such as lingual lipase and lysozyme, may be more abundant in stimulated saliva.
7. Immunological factors: the immune-related components in saliva, including immunoglobulins (e.g., IgA) and antimicrobial peptides, may be influenced by stimulation. Increased saliva flow during stimulation can enhance the presence of these immune factors, contributing to oral defense mechanisms.

It is also important to note that a variation exists among individuals and the aforementioned differences may not be universally applicable. Additionally, the specific differences in salivary composition between stimulated and unstimulated states can be further influenced by factors like oral health, general health status, drugs and nutritional habits.

Therefore, regardless of the method or type of saliva sample chosen, the procedures used to collect samples should be standardized as much as possible.

Saliva can be collected using four different methods: the passive drool method, the spit method, the suction method and the absorbent method [74,75].

The passive saliva collection method is considered the gold standard by many researchers [75] as it avoids any type of bias, such as reflex stimulation. Considering its ease of use, this method appears to be the most common for saliva collection in experimental studies.

Although there are no standardized procedures to reduce variability in saliva collection, there are several guidelines and considerations that ensure the least interference and greatest reproducibility of the collected specimens [76].

Saliva should be collected in the morning, 2 h after waking up to minimize the circadian rhythms’ influence, preferably after fasting or at least 2 h after eating and/or drinking [77]. Oral hygiene procedures should be performed at least 1 h before collection. Subjects should rinse their mouth with tap water for at least 30 s to remove desquamated epithelial cells, microorganisms and remnant food and drinks. Moreover, they should rest for 5 min before the collection to avoid sample dilution. Blood-contaminated samples must be rejected [77].

The total time required to collect saliva samples should be recorded in order to obtain the volume measured over time (production *per* unit of time, mL/min). Low flow rates are an indication of salivary gland pathological conditions or of specific drug use.

Saliva samples should be stored immediately after saliva collection at −20 °C or −80 °C, depending on the analyte being tested, until the analysis is performed [76].

At the time of saliva collection, several factors must also be considered that influence its composition and its flow rate, such as a high inter-individual and intra-individual variability or aging, which can induce hyposalivation [78]. In fact, the use of drugs and various pathological conditions, including diabetes mellitus, CKD, and liver and autoimmune diseases, can determine alterations in salivary components [79]. Other factors are body mass index, lifestyle, and, in particular, smoking or elevated use of caffeine, which appear to have strong effects on the salivary proteome pattern [76].

## 6. Impact of Oral Diseases on Oral Microbiota Composition

Dysbiosis of microbial communities in the gut and in the mouth precedes many oral and systemic diseases and can induce the breakdown of innate barriers and immune dysregulation. Moreover, this impairment of gut and oral microbiota can stimulate pro-inflammatory signaling.

The oral and maxillofacial regions present various anatomical structures that act as ecological niches for bacterial colonization [80]. The latter is influenced by nutrients availability, host immune system, oxygen content and temperature [24]. 

The oral microbiota has multiple stages of maturation, acquiring Streptococcus as pioneer colonizers before being populated by other organisms (Table 2) [81]. Although there is a wide inter-individuality in colonizing species, a ‘core taxa’ of oral microbes has been identified and it includes Bacteroidetes, Firmicutes, Actinobacteria, Proteobacteria, and Fusobacteria [82]. 

In general, *Streptococcus mitis* is found in the buccal mucosa, *Streptococcus salivarius* is found in the saliva and on the dorsal tongue and *Streptococcus sanguinis* is found on the tooth surface. These bacteria modify the environment through pH modulation, nutrients availability, and other factors, creating conditions for subsequent microbial colonization. Therefore, more complex bacterial communities are developed and a homeostatic balance of microorganisms within their respective niches is established. Although the genus Streptococcus dominates several oral surfaces, there are other bacterial species implicated in microbial homeostasis: Neisseria, Haemophilus, Corynebacterium, Rothia, Actinomyces, Prevotella, Capnocytophaga, Porphyromonas, and Fusobacterium phylotypes [83]. The bacterial genus mostly associated with oral cavity infections is *Streptococcus* spp. The oral cavity of a newborn is rapidly and primarily colonized by bacteria such as *Streptococcus salivarius*. During the first year of life, with the eruption of teeth, the colonization continues with *Streptococcus mutans* and *Streptococcus sanguinis*. Later, the gingival area becomes the ideal habitat for anaerobic bacteria belonging to Bacteroides and Spirochetes, which are generally associated with periodontal diseases. In addition to microbial infections that cause halitosis, gingivitis and periodontitis, other viral infections (herpesvirus) and oral fungal infections can frequently occur [84].

Among oral diseases, the most common are dental caries and periodontitis. Dental caries represents a prevalent chronic illness, characterized by the degradation of hard dental tissues from bacterial acids, which results in decay and loss of tooth structure. Its main etiological agent is *Streptococcus mutans*, which has the ability to resist higher levels of oxidative stress, thus changing the homeostatic balance of the oral microbiota [85]. However, recent studies examined other bacteria (present also in the gut) that can be involved in the pathogenesis of carious lesions [86]. Carious lesions initially caused by *Streptococcus mutans* tend to be fed by microbes belonging to Lactobacillus, Propionibacterium, the genus Atopobium and *Scardovia wiggsiae*. The latter has been strongly associated with severe early childhood caries (S-ECC) [87].

Periodontitis, while sharing the same dysbiotic organization of caries, follows different pathways and mechanisms of etiopathogenesis and progression [88]. 

Socranksy and co-workers described the so-called “red complex”, which includes *Porphyromonas gingivalis*, *Tannerella forsythia* and *Treponema denticola* [88], and they claimed that periodontitis is best represented by complexes of bacteria rather than by a single etiologic agent [89]. Upon primary infection of the dental pulp, in addition to the mentioned species, a significant level of *Enterococcus faecalis* was also observed. However, with root canal treatment and retreatment, elevated levels of *Enterococcus faecalis*, *Filifactor alocis*, *Pseudoramibacter alactolyticus*, *Parvimonas micra*, *Propionibacterium propionicus*, *Streptococcus constellatus* and *Streptococcus anginosus* have been detected [90]. 

Several studies have investigated whether the correlation between microbial dysbiosis in the oral cavity and in the gastrointestinal tract can trigger systemic diseases. When oral changes occur, the microbial balance is altered, raising the levels of bacterial species that favor the pathogenesis of various oral diseases, including dental caries, periodontitis and endodontic infections. 

In periodontitis disease, *Porphyromonas gingivalis* [91] may represent one of the clearest connections between oral and gastrointestinal dysbiosis. In fact, the oral administration of *Porphyromonas gingivalis* significantly increases endotoxemia and reduces the mRNA expression of occludin tight junction proteins (ZO-1) in the small intestine [92,93]. Furthermore, even a single oral administration of *Porphyromonas gingivalis* can increase the prevalence of Bacteroidetes while, in the meantime, it can decrease the abundance of Firmicutes [93]. In the GI system, the effect of *Porphyromonas gingivalis* is also magnified, despite its low abundance, changing the expression of tight junction proteins [93]. Moreover, its presence in the gut microbiota has been linked with inflammatory systemic diseases [12,13,94,95]. Therefore, the oral cavity and the gastrointestinal system have a close connection, which is also reflected in their specific microbiomes. This natural interconnection suggests potential routes for bacterial transfer.

Two hypotheses have emerged for the transmission of oral bacteria to the gut:

(i) the haematogenic route, whereby oral bacteria systematically circulate until they colonize the gastrointestinal mucosa;
(ii) the enteral route, in which bacteria from the oral cavity, via the stomach, reach the intestine.

The human body possesses several defense mechanisms and barriers against microbes, including their neutralization through gastric acidity. However, some mechanisms can impair these barriers (for example, the use of antibiotics alters the gut microbiota composition). Evidence for this comes from inflammatory bowel diseases (IBDs), which are known to encode antibiotic-resistant genes [96], thus paving the way for the colonization of the gastrointestinal tract. Furthermore, patients with achlorhydria, commonly associated with the long-term use of proton pump inhibitors, present high levels of oral bacteria in their gastrointestinal system [97]. Among these, there is also *Porphyromonas gingivalis*, which is known to be acid-resistant [98]. 

Regardless of the route of transmission, evidence suggests that more than half of the bacterial species present in the gastrointestinal system undergo an oro-intestinal translocation, even in the absence of pathology. Among oral bacteria that can be found in the gut of patients with gastrointestinal diseases, there are members of the genera Staphylococcus, Porphyromonas, Veillonella, Fusobacterium, Actinomyces and Parvimonas [97].

Gut dysbiosis has been observed in nephropathic patients [99] and many studies have also suggested an implication of the oral microbiome [100] in CKD pathogenesis. In fact, the oral microbiome, particularly the salivary microbiome, is altered in CKD patients and it is characterized by an increase in Lautropia, Pseudomonas and Neisseria and a decrease in Actinomyces, Prevotella 7, Veillonella, Haemophilus and Trichococcus [101]. Moreover, the last two have a negative association with estimated GFR (e-GFR) [101]. In addition, periodontal pathogens are increased in CKD patients undergoing hemodialysis [102]. Concurrently, the potential impact of the salivary microbiome on the onset and progression of diabetes mellitus and arterial hypertension was evaluated and it was found that these two diseases are correlated with CKD. Patients with concomitant diabetes mellitus show a decrease in the bacterial diversity of the gut and salivary microbiome [102,103,104,105] and higher levels of *Porphyromonas gingivalis*, *Tannerella forsythia,* and *Filifactor alocis* [106]. In contrast, patients with arterial hypertension have a higher concentration of certain pathogenic oral species in the oral plaque, such as *Actinobacillus actinomycetemcomitans* [107].

The kidneys are a frequent target of systemic immune diseases [108] and the human microbiome is responsible for the induction, development and modulation of immune responses [109]. Focusing on a few altered bacterial taxa, the phylum Actinobacteria and its genus Actinomyces are drastically decreased, as well as Prevotella 7, which plays an important role in CKD pathogenesis because it is a proteolytic bacterium that can break down proteins and peptides into amino acids [110], reducing the production of short-chain fatty acids (SCFAs).

It was discovered that immunoglobulin G (IgG) levels are negatively associated with Pseudomonas abundance [111]. IgG is an important factor for humoral immunity; lower serum levels of IgG are associated with a higher percentage of CKD, lower e-GFR and poor renal outcome [13,112]. This negative association between IgG and Pseudomonas indicates the involvement of the salivary microbiome in the regulation of immunity in CKD patients. In contrast, the taxa Lautropia is increased in CKD patients. The enrichment of salivary Lautropia could indicate an unhealthy state in the human oral cavity, as previous studies have shown their increase in various pathological conditions. For example, patients with erosive lesions due to oral lichen planus have a higher level of Lautropia compared to those without erosive lesions and healthy subjects [113]. In fact, Lautropia can be used as a diagnostic biomarker for patients with Barrett’s esophagus [114] and hepatitis B [115]. On the other hand, the decrease in Trichococcus in CKD patients could be associated with an unhealthy state of the oral cavity. A previous study demonstrated a decrease in Trichococcus in pediatric patients with obstructive sleep apnea [116]. However, considering the therapeutic front, further multicenter studies must be performed to correlate the salivary microbiome with the gut microbiome using intestinal permeability markers, inflammatory markers, epigenetic factors and biomarkers for renal function. Therefore, future clinical trials are necessary to better understand the salivary microbiome as a potential diagnostic biomarker and to investigate its diagnostic and therapeutic value in CKD patients. It was hypothesized that salivary microbiome transplantation could replace the therapeutic method of fecal microbiome transplantation in CKD [116].

Lactoferrin (Lf) is one of the components of saliva, along with hormones, peptides, organic and inorganic compounds (Fe^3+^, Mg^2+^, Na^+^, K^+^, Ca^2+^, Cl^−^, HPO_4_^2−^, and HCO_3_^−^), and enzymes [117]. It is a glycoprotein capable of chelating two iron atoms *per* molecule and has an anti-inflammatory and antibacterial function together with lysozyme, mucins (MG1 and MG2), IgA, IgM, IgG, alpha-amylase and organic compounds [117,118,119,120,121]. Saliva contains an Lf concentration of approximately 20 µg/mL and this value is altered in subjects with oral diseases [122,123]. As in the intestine, a free iron excess in the oral cavity stimulates microbial multiplication, synthesis of reactive oxygen species (ROS), inflammatory processes, pigment formation and the occurrence of black stains [121,124,125]. Iron is the most important element for the development of all living cells and for microbial virulence [121,126,127]. It was also reported that an overload of free and available iron in saliva is critical for the transition of bacteria from the planktonic into the sessile state in biofilms, which characterizes ineradicable oral infections [124]. Some bacteria, such as *Streptococcus mutans* and *Prevotella intermedia*, take advantage of free iron increase for their multiplication, amplifying the severity of gingivitis and periodontal diseases. 

All these mechanisms contribute to the onset of destructive inflammatory processes caused primarily by Lf deficiency, but these processes are also caused by an excess of available iron and an increased bacterial colonization [128,129].

**Table 2:** Changes in the oral microbiota according to different physiological and pathological conditions. (A) Impact of age on the oral microbiota. (B) Oral microbiota changes in oral diseases compared to healthy subjects. (C) Oral microbiota changes in NCDs. Abbreviation: A, Actinomyces; NCDS, chronic non-communicable diseases; S, Streptococcus.

A	**Phase of life**	**Age-related changes in Oral Microbiota**	**Reference**
	Newborn	*Streptococcus* (*S. salivarius* is the pioneer and then *S. sanguinis*, *S. peroris*, *S. lactarius*), *Actinomyces*	[82]
	Child (after 1 year of life)	Streptococcus (*S. mutans*), *Granulicatella*, *Actinomyces* (*A. odontolyticus*), *Fusobacterium*, *Abiotrophia*	[82]
	Adult	*Streptococcus*, *Lactobacillus*, *Bifidobacterium*, *Neisseria*, *Haemophilus*, *Corynebacterium*, *Rothia*, *Actinomyces*, *Prevotella*, *Capnocytophaga*, *Porphyromonas*	[83]
	Elderly	Increase in *Prevotella*, *Veillonella*, *Streptococcus*, *Candida*	[82]
B	**Presence of healthy oral cavity or oral diseases**	**Changes in Oral Microbiota related to oral cavity health**	
	Healthy subjects	*Streptococcus*, *Lactobacillus*, *Bifidobacterium*, *Neisseria*, *Haemophilus*, *Corynebacterium*, *Rothia*, *Actinomyces*, *Prevotella*, *Capnocytophaga*, *Porphyromonas*	[83]
	Periodontal Diseases	Increase in *Porphyromonas gingivalis*, *Tannerella forsythia*, *Treponema denticola*	[84] [85]
	Caries	Increase in *Streptococcus mutans*, *Lactobacillus*, *Propionibacterium*, *Atopobium genera*, *Scardovia wiggsiae*	[86] [87]
	Root infections	Increase in *Enterococcus faecalis*, *Filifactor alocis*, *Pseudoramibacter alactolyticus*, *Parvimonas micra*, *Propionibacterium propionicus*, *Streptococcus constellatus*, *Streptococcus anginosus*	[90]
C	**NCDs**	**Oral microbiota changes in NCDs**	
	CKD	Increase in *Lautropia*, *Pseudomonas*, *Neisseria* and decrease in *Actinomyces*, *Prevotella*, *Veillonella*, *Haemophilus*, *Trichococcus*	[92]
	Gastrointestinal diseases	Increase in *Staphylococcus*, *Porphyromonas*, *Veillonella*, *Fusobacterium*, *Actinomyces*, *Parvimonas*	[93]
	Diabetes mellitus	Increase in *Porphyromonas gingivalis*, *Tannerella forsythia*, *Filifactor alocis*	[13]
	Arterial hypertension	Increase in *Actinobacillus actinomycetemcomitans*	[13]

## 7. Pharmacological Treatment of Oral Dysbiosis

In order to avoid specific dysbiosis conditions of the oral cavity, physicians try to make limited use of broad-spectrum antibiotics, which can alter the entire microbial flora, incentivizing the use of a targeted therapy [130].

Broad-spectrum antimicrobial mouthwashes such as chlorhexidine are often used to control dysbiosis. However, a new decapeptide called KSL (KKVVFKVKFK-NH2) demonstrated significant antimicrobial effects through the inhibition of biofilm formation. This peptide has also shown antimycotic properties against Candida albicans.

Antimicrobial peptides may therefore prove to be an effective approach in restoring oral health [131].

Using saliva sampling, it has been shown an individual predisposition to caries development. Subjects that presented increased levels of lipid breakdown products, decreased salivary pH and low salivary microbial diversity with a prevalence of saccharolytic microbes were more prone to develop caries. In contrast, individuals with increased salivary pH, reduced lysozyme activity and a prevalence of proteolytic microorganisms were predisposed to periodontal disease and gingival inflammation [132].

*Streptococcus mutans* has two key virulence factors: the surface adhesin protein PAc (Antigen I/II, P1) and glucosyltransferases (GTF) used to generate glucans from sucrose [133]. An attempt was made to develop a targeted therapy against these virulence factors in oral *Streptococcus mutans* using a monoclonal antibody, and this study showed promising results [134]. The rate of caries was reduced after administration of polyclonal IgG antibodies against GTF and glucan binding protein (GBP) [135].

Individuals with a low salivary pH and a cariogenic ecotype may benefit from treatment with *Streptococcus dentisani* [136]. This novel strain is cultured from the dentition of caries-free individuals, and it seems to increase the pH in the oral environment through the breakdown of arginine with subsequent ammonia production [137]. Furthermore, it was discovered that *Streptococcus dentisani*-derived supernatants inhibit the growth of many oral pathogenic microorganisms, including *Streptococcus mutans*, *Streptococcus sobrinus*, *Fusobacteriun nucleatum,* and *Prevotella intermedia*, showing structural changes in the cell wall [138].

## 8. The Possible Link between Oral Dysbiosis and Gut Dysbiosis and Its Influence on CKD Onset and Progression

**Figure 2:**
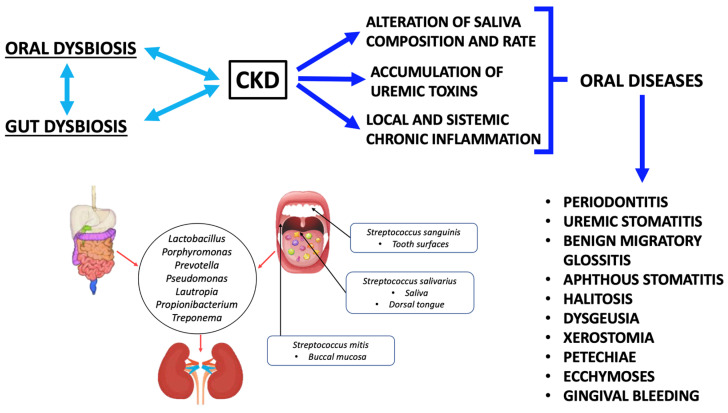
The link between oral dysbiosis, gut dysbiosis, and chronic kidney disease. Abbreviations: chronic kidney disease, CKD.

Several studies showed the presence of alterations in saliva composition and oral microbiota in CKD patients. Saliva is a unique biological exocrine excretion, which is composed of hundreds of different proteins and thousands of peptide sequences [116]. The composition and the correct amount of saliva are crucial to preserve the health of the oral cavity [139]. The total amount of saliva produced *per* day is about 500–1500 mL [140]. There are similar functions between salivary glands and renal tubular epithelial cells: in fact, the saliva composition is more similar to urine than blood plasma. Furthermore, a study showed that the saliva composition in CKD patients changed in association with an increase in urea, creatinine, calcium, sodium, potassium, phosphorus, bicarbonate and phosphate blood levels compared to the control group [141,142,143,144]. These modifications showed a direct association between blood and salivary alterations [144]. Therefore, a mutual correlation between CKD and oral diseases is evident. In fact, a low-grade chronic inflammatory status, a low salivary flow rate and its impaired composition, and oral microflora dysbiosis, contribute to inducing or worsening CKD. At the same time, the presence of CKD is a possible cause of oral disease onset (Figure 2).

In this regard, a study conducted by Trzcionka underlined a correlation between CKD and oral diseases, especially caused by saliva deficiency [145]. Moreover, in end-stage renal disease (ESRD), oral alterations such as benign migratory glossitis, aphthous stomatitis, ecchymoses, xerostomia, petechiae and gingival bleeding can be detected [145,146,147,148,149,150,151]. CKD patients often have halitosis [152], dysgeusia and an increased risk of periodontal disease [153]. Halitosis can be caused by urea increase in saliva secretions. In fact, urea is converted into ammonia (NH_3_) by oral enzymes [142]. Regarding the buffer capacity of saliva, a study conducted by Trzcionka et al. observed that CKD patients under conservative therapy had a low buffer capacity compared to hemodialysis (HD) patients [145].

Previous studies demonstrated that both CKD patients under conservative therapy and patients on renal replacement therapy (RRT) have impaired saliva composition, and they never restore physiological metabolite levels [154]. Despite that, HD can help CKD patients by enhancing the salivary flow rate. There are different hypothesizes that explain this phenomenon:

(i) HD can restore the intravascular volume because of an ultrafiltration mechanism. The latter causes a high gland perfusion able to stimulate saliva production [155].
(ii) HD treatment corrects blood concentrations of electrolytes and bicarbonate and reduces serum creatinine and urea levels. This correction induces a higher production of saliva, and it may also happen after the first hemodialysis session [156].
(iii) HD session reduces arterial blood pressure. This phenomenon could favor the sympathetic activity of salivary glands and, therefore, the production of saliva [142].

Regarding peritoneal dialysis (PD), a study conducted on pediatric PD patients by Freitas-Fernandes demonstrated an increase in salivary creatinine in PD patients compared to subjects with normal renal function (control group) matched for age. The authors proved that PD could not reestablish adequate levels of salivary creatinine [154].

Another study showed that children with CKD had higher levels of dental calculus and lower levels of dental caries, probably due to an increase in salivary urea concentration and a change in pH [157,158].

A case-control study monitored the salivary concentration of calcium and phosphorus in adult CKD patients [142]. The authors demonstrated that there were higher salivary concentrations of calcium, phosphorus and potassium in CKD patients compared to the control group with normal renal function. These data are probably due to alterations in calcium–phosphorus metabolism induced by CKD.

The most efficient system to assess the GFR is the creatinine clearance [159]. Furthermore, the urea blood concentration represents an indirect biomarker for the monitoring of renal function [160]. 

In order to monitor the disease severity, CKD patients need to undergo blood tests repeatedly. To avoid this invasive procedure [161], a possible alternative is to analyze salivary compounds. Currently, they seem to be effective biomarkers that can help monitor the stage of several diseases [162,163]. Their advantages are low cost, easy collection and non-invasiveness [164]. 

Other factors that influence oral microflora composition are uremic toxins (UTs). UTs are directly related to the CKD stage and severity. Their increase can impact on the oral environment, generating a link between oral dysbiosis and CKD [146]. UTs accumulation is a consequence of decline in renal function. 

Some UTs are gut-derived metabolites [165] and the main ones include: 

(i) phenols, comprising phenyl sulfate, p-cresol, p-cresol sulfate (PCS), phenylacetic acid, sulfate, p-cresyl (PC) and p-cresyl glucuronide (PCG). These compounds are mostly generated by the tobacco consumption and the ingestion and catabolism of tyrosine and phenylalanine through intestinal bacteria. Moreover, PCS is the main circulating metabolite of p-cresol [166].

(ii) Indoles, including indoleacetic acid (IAA) and indoxyl sulfate (IS). These compounds are derived from tryptophan degradation by gut bacteria. IAA is consequently sulfated in the liver into IS [167].

(iii) Amines and polyamines. These compounds are also derived from gut microbial metabolism. The most important amine is trimethylamine N-oxide (TMAO), produced by quaternary amine metabolism, such as betaine, choline/phosphatidylcholine, and L-carnitine [168]. Polyamines include spermidine, spermine, putrescine and cadaverine [169].

Recent studies by Noce et al. demonstrated that CKD progression is also related to oral dysbiosis [12,170,171]. In particular, three oral pathological conditions (oral infections, periodontitis and uremic stomatitis) may induce and exacerbate systemic chronic inflammation [172]. In turn, the latter causes cell-mediated immunity suppression. This phenomenon partly explains the susceptibility of CKD patients to infections [146]. In fact, the oral microbiome of CKD patients could be colonized by enterobacteria in the periodontal pockets [173], thus favoring the systemic inflammation. Moreover, a lot of these pathogenic bacteria (like Enterobacteriaceae), have an antibiotic-resistant phenotype. For example, these bacteria have chromosomal genes that codify different proteins, like antibiotic-inactivating enzymes and proteins involved in non-enzymatic pathways (such as mechanisms that regulate cell permeability, efflux pumps and target molecule modifications) [174]. 

Oral dysbiosis in CKD patients provokes oral bacterial translocation into the bloodstream during different daily activities, such as tooth brushing, and during invasive dental procedures. Therefore, oral bacteria may become opportunistic infectious agents in different body sites, like the peritoneum [175]. 

At the same time, CKD seems to interfere with the gut and oral microbiota composition, generating a harmful dysbiosis either in the gut or oral cavity [176]. 

In 2019, Olsen and Yamazaki suggested that oral dysbiosis can affect the gut microflora, causing systemic dysfunctions [177].

Moreover, an in vivo study showed that the administration of 109 CFU of live *Porphyromonas gingivalis* (strain W83), a relevant periodontal pathogen, to C57BL/6 mice, twice a week, for 5 weeks, influenced not only the gut microbiota but also the mucosal permeability, gut physiological functions and bacterial-derived toxin concentrations in the bloodstream [92]. Regarding the gut microflora, a decrease in the proportion of the phylum Firmicutes and RoRγ t gene expression and an increase in the proportion of the phylum Bacteroides were observed [93]. 

Lately, other studies demonstrated that oral bacteria can cause an imbalance in the gut microbiota and the immune system; these bacteria include Streptococcus, Fusobacterium and Staphylococcus [178]. Moreover, the M1/M2 macrophage ratio in the small gut seems to increase because of the alteration in oral microflora. On the contrary, it was shown that Lactobacillus and other probiotic bacteria can suppress the M1/M2 macrophage ratio, inducing an anti-inflammatory action [179]. 

As previously described, there seems to be a mutual relationship between CKD and gut dysbiosis. In fact, gut dysbiosis increases the risk of developing CKD and its comorbidities (like cardiovascular diseases, arterial hypertension, diabetes mellitus, etc.), especially in elderly people [180]. This link is closely related to the consequences of gut dysbiosis, characterized by alterations in several metabolic pathways and the immune system [181]. The first scientific evidence for this connection was found in the study conducted by Simenhoff et al. [182]. The authors showed that gut dysbiosis should increase the pre-existent renal damage through several mechanisms including:

(i) gut bacteria produce metabolites, such as TMAO, IS, PCS and phenylacetylglutamine (PAG), with a toxic action against the kidneys and the cardiovascular system [99].
(ii) In CKD patients, there are alterations in the gut microflora characterized by an increase in pathogenic species. In these patients, alterations in gut permeability can be observed. These permeability alterations allow the translocation of endotoxins into the bloodstream. This phenomenon worsens the systemic low-grade inflammatory state, accelerating the CKD progression [183].

On the other hand, CKD itself impacts on gut microbiota composition [180]. In particular, CKD nutritional and pharmacological treatments should increase gut dysbiosis [184]. One concern is the negative effect caused by the use of drugs. In particular, antibiotics can alter the gut microflora, while other medicines, such as ion exchange resins, phosphorus binders and iron supplements, can slow down the physiological intestinal transit [185,186,187,188,189].

In fact, one of the most common complications of CKD is iron deficiency anemia (IDA). To treat IDA, oral iron therapy is often administered to pre-dialysis patients. The gut is a key modulator of iron homeostasis and iron oral supplementation is an effective option to replenish iron stores. However, adverse effects on the gut microbiota have been reported, like an increased risk of gut inflammation [190,191]. 

**Figure 3:**
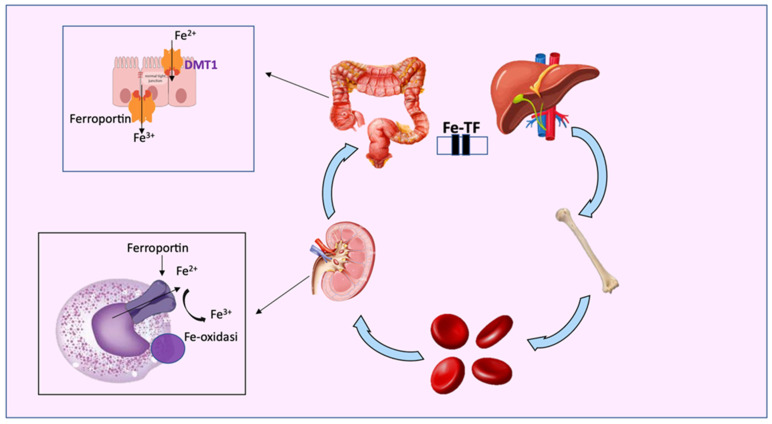
Iron metabolism: transferrin (TF) transports free iron, both the ferrous (Fe^2+^) and ferric forms (Fe^3+^), to tissues and organs, such as the bone marrow and liver. Free iron is active after dissociation from TF, while excess intracellular iron is exported from ferroportin (FPN) or stored as Fe^3+^ in ferritin. Iron is also released by metal divalent conveyor 1 (DMT1).

Recently, it was shown [120] that circulating iron deficiency is not associated with a true lack of iron in the body but with its delocalization (Figure 3). In physiological conditions, iron is absorbed daily by the duodenal enterocytes (1–2 mg/day) and modulated by ferritin which, after sequestering iron, releases it back to the cell via ferroportin (Fpn), the only protein capable of exporting iron from the cells to the circulation. This mechanism also occurs in other cells, including epithelia in the oral mucosa. In the absence of Fpn, iron cannot be exported and remains accumulated within the cell. Furthermore, the synthesis of Fpn is under the control of IL-6 (a pro-inflammatory cytokine). An increased concentration of IL-6 inhibits the synthesis of Fpn, inducing the accumulation of iron in the tissues and, at the same time, its deficiency in the bloodstream [118,120]. In other words, iron supplementation results in a lack of the element’s absorption, which then reaches the colon, where it is potentially available for the gut microbiota. Free iron can stimulate the virulence of pathogenic bacteria residing in the gut and it can contribute to the development of a proinflammatory oxidative environment [190], which can directly affect intestinal epithelial integrity [192]. Consequently, an impairment of the gut barrier could lead to an increased exposure of the host to the endotoxins. These mechanisms can induce systemic microinflammation in CKD patients and local renal immune cell responses, accelerating cardiovascular comorbidities and the progression of renal failure [99,193,194].

Oral iron administration causes a reduction in the number of species, like Lactobacillaceae and Bifidobacteriaceae, which are generally beneficial. Therefore, the simultaneous intake of these families of probiotic and/or prebiotic bacteria can counteract the side effects of iron administration and can contribute to the maintenance of these beneficial strains in the colon. Prebiotic fibers can increase the number of Bifidobacteriaceae and decrease the pH in the colon [195]. Similarly, natural forms of iron, such as Lf, could be good candidates to replace the current oral iron supplements [187,196]. When the Lf physiological concentration in the saliva is restored with one or more daily administrations, IL-6 synthesis decreases and, subsequently, Fpn function is resumed. In other words, Lf prevents and treats inflammation, the main cause of iron overload, and infection in the mucous membranes, thus restoring Fpn physiological synthesis and reversing iron homeostasis disorders [197,198].

Therefore, pharmacological treatments contribute to altering the intestinal surface, in relation to a reduced production of SCFAs (derived by saccharolytic fermentation). In fact, SCFAs physiologically protect the gut mucosa against damages [165]. 

Increased mucosal permeability generates an access point for bacterial products of intestinal origin (i.e., DNA fragments of intestinal aerobic and anaerobic pathogens). The presence of these products in the bloodstream activates innate immunity and inflammatory pathways and increases cardiovascular risk [185,186,188,190,199,200,201,202].

An elevated UTs concentration in the blood increases the gut permeability. This impairment of the intestinal barrier induces gut colonization of bacteria, some of which can express ureases and uricase enzymes, which convert urea into ammonia. Ammonia raises the gut pH, influencing the growth of pathogenic bacteria that can subsequently cause dysbiosis [203]. The presence of UTs in combination with an impaired gut permeability generates a worsening of inflammation and oxidative stress [183].

A study conducted by Vaziri et al. revealed that CKD patients from several ethnicities, differed of 190 bacterial operational taxonomic units (OTUs), in particular, Pseudomonadaceae, Actinobacteria, Firmicutes (mainly Clostridia) and Proteobacteria families were changed compared with the control group. Another analysis confirmed this finding. In fact, different subjects showed an increment of pathogenic bacteria in the gut, especially new micro-florae that express enzymes involved in the conversion of aromatic amino acids and in the production of IS or PC [204,205].

In another study conducted on 24 CKD patients from different ethnicities, it was demonstrated that CKD patients express:

(i) twelve of the nineteen bacteria families with urease activity, including Clostridiaceae, Dermabacteraceae, Halomonadaceae, Methylococcaceae, Alteromonadaceae, Cellulomonadaceae, Pseudomonadaceae, Xanthomonadaceae, Enterobacteriaceae, Moraxellaceae, Polyangiaceae, and Micrococcaceae;
(ii) three families with tryptophanase activity, including Verrucomicrobiaceae, Clostridiaceae, and Enterobacteriaceae;
(iii) five families with uricase activity, including Dermabacteraceae, Micrococcaceae, Cellulomonadaceae, Xanthomonadaceae, and Polyangiaceae.

These patients also registered a reduction in the Prevotellaceae and Lactobacillaceae families, which are involved in the protective processes of the gut mucosa [206].

Furthermore, CKD patients show a relevant susceptibility to oral diseases, especially due to the Enterobacteriaceae family, which also represents etiological agents of dialysis-associated and nosocomial infections [175]. Enterobacteriaceae species are particularly pathogenic in ESRD patients; in fact, they are responsible from 10 to 12 percent of all peritoneal dialysis-associated peritonitis [207]. A study that involved PD patients showed that they presented more microbial counts and Enterobacteriaceae compared to the controls. The authors also observed a high diversity of Enterobacteriaceae species. In fact, the controls presented only three species, while the PD patients presented eight species. In particular, *Raoultella ornithinolytica*, a histamine-producing aquatic-commensal enterobacteria, was significantly present in the oral cavity of PD patients, but it was absent in the oral cavity of the controls. In detail, the colonization of this bacteria depended on the age, sex and ethnicity of the participants. *Raoultella ornithinolytica* can rarely survive in human saliva and several studies demonstrated [175] that this species is responsible for primary peritonitis in humans [175,208].

In conclusion, the presence of UTs, the alterations of metabolic pathways, high levels of ammonia and urea and high pH, are involved in the alteration of the oral environment as well as of the gut environment [146,209].

## 9. Pharmacological and Nutritional Treatment of Gut Dysbiosis in CKD

As previously described, there is a strict correlation between gut dysbiosis and CKD. Therefore, an amelioration of one of them seems to influence both positively. In fact, a study conducted by Ramezani et al. showed that a change in the gut microbiota can influence CKD pathogenesis [201]. 

Several studies were conducted with the aim of finding a new possible therapy for CKD gut dysbiosis. One study was based on the administration of specific prebiotics [210]. A prebiotic is a compound used against host microorganisms and is characterized by healthy properties [211]. These substances should be inulin-type fructans (oligofructose, fructo-oligosaccharides and inulin), galactans (galacto-oligosaccharides), polyunsaturated fatty acids (PUFAs), polyphenols and conjugated linoleic acids [210,212]. Among them, an element that can have a beneficial role in CKD patients affected by gut dysbiosis is the resistant starch (RS), a α-linked glucose polymer that is not hydrolysable in the human small gut [213]. Two studies conducted on healthy and CKD animal models, fed with a RS-supplemented diet, highlighted a reduced plasma urea concentration [210,214]. Vaziri et al. demonstrated that, in mice models with adenine-induced CKD, there was an improvement in creatinine clearance, serum creatinine, interstitial fibrosis and renal inflammation, after a diet containing 59% high-amylose maize starch [99]. To date, the benefits of RS have not been confirmed in human studies on CKD patients. 

Therefore, dietary fibers, including RS, are capable to positively modify the gut microbiota because they represent a nutritional substrate for saccharolytic bacteria [210]. On the contrary, there are some substances that can decrease the gut microbial balance, such as antibiotics (like amoxicillin) or alcohol [215,216]. 

Other oral food supplements used for gut dysbiosis are probiotics, that are, “live microorganism that show beneficial effects on the health of the host” [217]. Among the different species, *Saccharomyces cerevisiae* is the most used. There are also other types like Lactobacillus (*L. john-sonii*, *L. sporogens*, *L. casei*, *L. plantarum*, *L. bulgaricus*, *L. delbrueckii*, *L. salivarius*, *L. rhamnosus*, *L. reuteri*, and *L. acidophilus)*, Bifidobacterium *(B. longum*, *B. bifi-dum*, *B. breve* (Yakult), *B. lactis*, *B. bifidus*, and *B. infantis*), Streptococcus (*S. thermophilus* and *acidophilus*), *Enterococcus* SF68, *Lactococcus lactis*, and *Escherichia coli* Nissle (serotype O6:K5:H1) [218]. 

Symbiotics are sometimes used to improve host conditions, not only in CKD patients but also in other pathological conditions. They are dietary supplements or food ingredients, composed of probiotics and prebiotics that work together for the health promotion. FOS/*Lactobacillus sporogens* and OAT fiber/*Lactobacillus plantarum* are actually used in clinical practice [219].

In conclusion, probiotics and prebiotics induce several beneficial effects, including the competitive exclusion of pathological bacteria in gut colonization, integrity and homeostasis of the gut, metabolism of primary to secondary bile salts, production of vitamins and SCFAs, regulation of gastrointestinal transit and neutralization of carcinogens or xenobiotics [220]. These positive changes could represent a good start for the gut dysbiosis treatment in CKD patients and a possible solution to ameliorate the host’s health.

## 10. Conclusions

Saliva composition monitoring could be a new, cheap, non-invasive and easy tool to diagnose and clinically evaluate oral and systemic diseases. From this perspective, it would be useful to standardize the saliva analysis method in order to apply it on a large scale.

Moreover, recent studies demonstrated a correlation between oral and systemic diseases and this connection is represented by gut and oral microbiota dysbiosis. Chronic degenerative NCDs, in particular, CKD, are characterized by gut dysbiosis and recent studies also highlighted the presence of oral dysbiosis in these pathological conditions.

In this context, it is important to promote the positive modulation of oral and gut microbiota in order to counteract dysbiosis with the administration of specific prebiotics, postbiotics and synbiotics.

## Figures and Tables

**Table 1:** Salivary biomarkers and tests currently in use.

Salivary Biomarker	Method	Pathological Condition	Reference
Alfa-amylase	Enzymatic colorimetric Chromolytic assay	Diabetes mellitus Renal diseases Physical exercise CVD	[27,28,29,30]
Glucose	Colorimetric assay Enzymatic colorimetric with guaiacol diazo derivative Luminescent method LC-MS GC-MS	Diabetes mellitus Arterial hypertension CVD Caries Periodontitis Obesity	[27,31,32,33,34,35]
Calcium	LC-MS GC-MS	Caries Periodontitis Arterial hypertension Alzheimer’s disease Parkinson’s disease Diabetes mellitus	[14,36,37,38,39]
Magnesium	LC-MS GC-MS	Caries Periodontitis Diabetes mellitus	[36,38,39]
Testosterone	ELISA CLIA LC-MS/MS Radioimmunoassay Different HPLC-MS/MS	Periodontitis Diabetes mellitus Obesity	[40,41,42,43,44,45,46]
C-reactive protein	ELISA Immuno-turbidimetric method EIA	Periodontitis Oral disorders CVD Pneumonia Metabolic disorders HIV CKD COVID-19 Rheumatic disease	[47,48,49,50,51]
Cortisol	Enzyme immunoassay Enzyme immunoassay ELISA ECLIA RIA	CVD Oxidative stress Physiological stress Metabolic syndrome Obesity	[27,41,52,53]
SARS-CoV-2 specific IgA	EIA ELISA designed for POC ELISA	COVID-19	[54,55,56]
SARS-CoV-2 antigen	Rapid Salivary Test (RST) based on the LFA Electrochemiluminescence (ECL)-based immunoassay	COVID-19	[57,58]
RNA SARS- CoV-2	rRT-PCR Colorimetric RT-LAMP assay Saliva-based, loop-mediated, isothermal amplification (LAMP) technology	COVID-19	[57,59,60,61]
Interleukines (IL-1β, IL-2, IL-4, IL-5, IL-6, IL-10)	ELISA Bead-based Xmap Bead-based flow cytometry	Lung cancer Graft-versus-host disease Mucositits HIV OSCC CVD Rheumatic diseases	[27,51,62,63,64,65,66,67]
TNFα	ELISA Bead-based xMAP Bead-based flow cytometry	Mucostitis Lung cancer HIV Tubercolosis OSCC CVD Rheumatic diseases	[27,51,62,66,67,68,69]
INFγ	ELISA Bead-based xMAP Bead-based flow cytometry	Lung cancer HIV Tubercolosis CVD	[27,62,67,69]
